# Recent Progress in Two-Dimensional Nanomaterials for Flame Retardance and Fire-Warning Applications

**DOI:** 10.3390/molecules29081858

**Published:** 2024-04-19

**Authors:** Weiliang Lin, Yao Yuan, Lulu Xu, Wei Wang

**Affiliations:** 1 Fujian Provincial Key Laboratory of Functional Materials and Applications, School of Materials Science and Engineering, Xiamen University of Technology, Xiamen 361024, China; weilianglin318315@163.com; 2 School of Chemical Engineering, University of New South Wales, Sydney, NSW 2052, Australia; lulu.xu1@unsw.edu.au; 3 School of Mechanical and Manufacturing Engineering, University of New South Wales, Sydney, NSW 2052, Australia

**Keywords:** graphene-like 2D nanomaterial, flame retardant, fire warning, mechanism

## Abstract

Graphene-like 2D nanomaterials, such as graphene, MXene, molybdenum disulfide, and boron nitride, present a promising avenue for eco-friendly flame retardants. Their inherent characteristics, including metal-like conductivity, high specific surface area, electron transport capacity, and solution processability, make them highly suitable for applications in both structural fire protection and fire alarm systems. This review offers an up-to-date exploration of advancements in flame retardant composites, utilizing pristine graphene-like nanosheets, versatile graphene-like nanosheets with multiple functions, and collaborative systems based on these nanomaterials. Moreover, graphene-like 2D nanomaterials exhibit considerable potential in the development of early fire alarm systems, enabling timely warnings. This review provides an overview of flame-retarding and fire-warning mechanisms, diverse multifunctional nanocomposites, and the evolving trends in the development of fire alarm systems anchored in graphene-like 2D nanomaterials and their derivatives. Ultimately, the existing challenges and prospective directions for the utilization of graphene-like 2D nanomaterials in flame retardant and fire-warning applications are put forward.

## 1. Introduction

Polymeric materials are prevalent in contemporary society, finding extensive use in packaging, construction, automotive, electronics, medical devices, aerospace, and various other industries. Nevertheless, the routine use of polymer materials poses significant safety risks, primarily stemming from their organic, frequently aliphatic structure. This structural characteristic leads to the propagation of fire when they are exposed to heat sources or open flames, ultimately culminating in fires that spiral out of control. Additionally, they have the potential to produce a significant amount of harmful smoke and toxic gases when undergoing burning. As a result, their extensive usage poses a substantial fire risk to lives and property, leading to an increased occurrence of fires and incurring immeasurable losses. In 1998, mainland China experienced 142,326 fires (excluding forest fires, grassland fires, and military fires), leading to direct losses of 1.44 billion yuan (approximately 173.5 million US dollars), 2389 fatalities, and 4905 injuries. In contrast, by 2022, the number of fires in China had surged to 825,000, leading to 2053 deaths, 2122 injuries, and direct property losses amounting to CNY 7.16 billion (approximately USD 1.001 billion) [1]. In 2019, global events such as the Notre Dame Cathedral fire in Paris, the “Black Summer” forest fire in Australia, and the Amazon rainforest fire led to substantial economic losses and environmental devastation [2]. Therefore, the development of effective fire protection strategies is crucial to mitigate and combat the damage caused by wildfires.

Fire prevention can be divided into two main aspects: structural fire protection and fire alarm protection. Structural fire protection is geared towards impeding the rapid propagation of a fire, ultimately minimizing damage to property and ensuring the safety of lives. Prevention strategies primarily involve the creation of highly flame-retardant materials. This is achieved either through the incorporation of flame retardants into the materials or through molecular and chemical modifications that introduce flame-retardant groups such as nitrogen and phosphorus [3,4,5,6,7]. These modifications serve to improve the flame resistance and smoke suppression of the materials, contributing to a more effective fire prevention system. Conversely, fire alarm protection delivers alerts to individuals before a fire breaks out or during its initial stages. Prevention efforts primarily revolve around the investigation of fire alarm systems, including infrared temperature alarm systems that respond to temperature variations and smoke alarm systems that react to particle concentrations in the air. When exposed to flame, smoke, or heat, the sensor activates, sending fire-warning signals in advance of a fire occurrence [8,9].

As is widely recognized, there are two primary strategies for imparting fire resistance to polymers: the introduction of flame-retardant elements (such as P, N, Si) through chemical reactions, or physical blending. From an economic standpoint, the prevalent preference lies in the utilization of the physical method, notwithstanding the requirement of traditional flame retardants for a comparatively elevated loading content to attain satisfactory flame retardance. Within the diverse range of nanomaterials, two-dimensional (2D) nanomaterials such as graphene, molybdenum disulfide, boron nitride, black phosphorene, and MXene show exceptional performance, presenting prospective substitutes for traditional flame retardants in polymer materials due to their low loading content [10]. It is widely acknowledged that adequately dispersed 2D nanosheets can instigate a “labyrinth effect”, elongating the airflow path within the polymer matrices. As a result, the ensuing high-quality chars significantly hinder the continued combustion of polymers. The development of graphene-like 2D nanomaterials has garnered significant interest and paved the way for a promising new approach to creating multifunctional flame-retardant polymeric materials.

Cutting-edge fire alarm protection systems play a crucial role in the implementation of fire safety measures. Specifically engineered to discern early indicators of potential fires, these systems expeditiously apprise occupants, facilitating prompt and essential actions in response. The systems employ various sensors and devices to identify indicators such as smoke, heat, or flames, triggering alarms and notifications, and their functionality is based on the principles of electrical resistance, phase/shape/color attributes, and the thermoelectric effect [11,12]. The unique characteristics of 2D nanomaterials have found widespread utility in fire alarm systems (FASs), where the incorporation of graphene-like 2D nanomaterials substantially reduces response times [13,14]. This enhancement facilitates quicker responses to potential fire incidents. The outstanding conductivity and expansive surface area of graphene-like nanomaterials make them ideal candidates for elevating FAS performance. The integration of graphene-like nanomaterials into FASs represents a significant stride in fire safety technology, underscoring the transformative potential of partial 2D layered nanomaterials in reshaping the landscape of fire detection and response.

Several published reviews have noted significant progress in the potential applications of 2D nanomaterials in fire retardancy or fire warning [15,16,17]. However, a comprehensive and critical review addressing structural fire protection and fire alarm protection has been lacking. This review conducts a comprehensive analysis of the design considerations related to fire retardants and fire-warning sensors, emphasizing their incorporation of graphene-like 2D nanomaterials. The exploration covers various facets, including their characterizations, modifications, performance, applications, and underlying mechanisms. Furthermore, this review meticulously examines and discusses potential strategies for the implementation of applications rooted in these graphene-like 2D nanomaterials.

## 2. Flame-Retarding and Fire-Warning Mechanisms

### 2.1. Combustion Characteristics of Polymer Materials

The combustion characteristics of polymer materials encompass a spectrum of behaviors crucial for assessing fire safety. This study meticulously investigates key parameters, including ignition temperature, flame spread, heat release rate, smoke and toxic gas emissions, char formation, and self-extinguishing properties [18,19,20,21]. When exposed to sufficient heat or fire, polymers undergo degradation, involving a series of intricate reactions such as backbone rupture, removal of side groups, and chain scission [22,23]. With the escalating heat supply from the ignition source, combustion persists, generating volatiles and smoke that enter the gas phase. Significantly, the critical region is positioned at the interface between the polymers and the flame, forming the phase boundary between the condensed and gas phases.

### 2.2. Gas-Phase and Condensed-Phase Flame Retardant Mechanism

Within the gas phase, chemical reactions mediated by flame retardants between oxygen and volatiles have the capability to extinguish the combustion of polymers. For flaming combustion the chain reaction of free radicals is a crucial factor, and interrupting it can lead to flame self-extinguishment. The free radical mechanism involves flame retardants that act in the gas phase to impede combustion, with antimony-halogen-containing flame retardants being a typical example [24]. Furthermore, during the combustion of flame-retardant polymers, a significant release of non-combustible gases occurs, diminishing the concentration of combustible gases or gases aiding combustion and thereby delaying flame spread. Flame-retardant polymers release non-combustible gases, diluting combustible concentrations and delaying flame spread. Compounds such as melamine-based ones release inert gases, aligning with typical gas-phase flame retardancy mechanisms [25,26,27,28]. Gas-phase flame retardants, although insufficient alone, are often combined with other types, playing a crucial role by providing time for reactions in the condensed phase.

The interaction between polymers and fire plays a pivotal role in combustion processes, influencing the slowing or interruption of combustion in the solid phase. This results in an accelerated polymer decomposition and the generation of increased carbon layers on the polymer surface. The protective efficacy of the carbon layer depends on its physical and chemical composition. When a substantial foam-like-structured carbon residue forms on the surface, this process is often referred to as expansion. Flame retardant systems with expansion typically incorporate phosphorus and nitrogen elements, adhering to the condensed phase mechanism during combustion. Furthermore, the incorporation of graphene-like nanomaterials, characterized by their layered structures and lamella-blocking effects, imparts various distinctive features. These features serve to hinder oxygen access, protract the heat transfer between interfaces, impede the escape of pyrolysis products, and facilitate oxygen mixing [29,30,31]. This phenomenon is commonly referred to as the “labyrinth effect”.

The flame-retardant properties of graphene-like 2D nano-materials stem from their ability to serve as barriers, exhibit catalytic effects, and undergo dehydration carbonization. Modified graphene-based materials, such as graphene oxide (GO) or reduced graphene oxide (rGO), can display catalytic properties due to the presence of functional groups or defects, which act as active sites for facilitating chemical reactions. Dehydration carbonization refers to the thermal decomposition of organic compounds in the absence of oxygen, leading to the formation of a carbonaceous char residue. This char layer acts as a protective barrier against combustion, crucial for flame retardancy by preventing fire spread. Additionally, graphene forms a layered structure within polymer matrices, creating a physical barrier to heat and flame propagation [32]. Incorporating thermally stable materials such as graphene, MoS_2_, and MXene into the polymer matrix enhances its barrier effect, thereby affecting the transfer of heat and pyrolysis products between the fire and the polymer matrix. When exposed to high temperatures and an oxygen-rich environment, MoS_2_ and MXene undergo transformation into metal oxides. The synthesis process during combustion is intense, resulting in the release of a significant amount of lattice oxygen and holes. Subsequently, active radicals are stabilized by these holes, facilitating the conversion of toxic CO into CO_2_ through a reaction with lattice oxygen. Additionally, the impact of the dispersion state of graphene-like 2D nanomaterials on improving the fire safety of polymer materials is significant [33]. Unfortunately, most graphene-like materials tend to agglomerate and re-stack, and their hydrophilic nature contributes to inadequate dispersion within polymer matrices [34]. Yuan et al. [35] have investigated the influence of graphene with poor/good dispersion on fire behavior in a polypropylene (PP) matrix. The results indicated that a low loading of well-dispersed graphene exhibited superior fire safety compared to its poorly dispersed counterpart. Therefore, various methods such as intercalation, exfoliation, and self-assembly are commonly employed to pre-treat layered nanomaterials before their incorporation into polymer matrices [36]. The subsequent sections will intricately explore the specific modifications and applications of graphene-like 2D nanomaterials.

### 2.3. Fire-Warning Mechanisms

The remarkable electrical conductivity of graphene-like 2D nanomaterials can be exploited for fire-warning applications. Integrating these nanomaterials into composite materials allows for the detection of heat or fire-induced structural changes or degradation through shifts in electrical resistance. By monitoring these changes in resistance, it is possible to develop a fire-warning system that detects the onset of a fire. Moreover, graphene-like 2D nanomaterials can be sensitive to changes in their environment, such as temperature and the presence of certain gases. This sensitivity can be used to trigger alarms or activate fire-suppression systems before a fire spreads.

**Figure 1:**
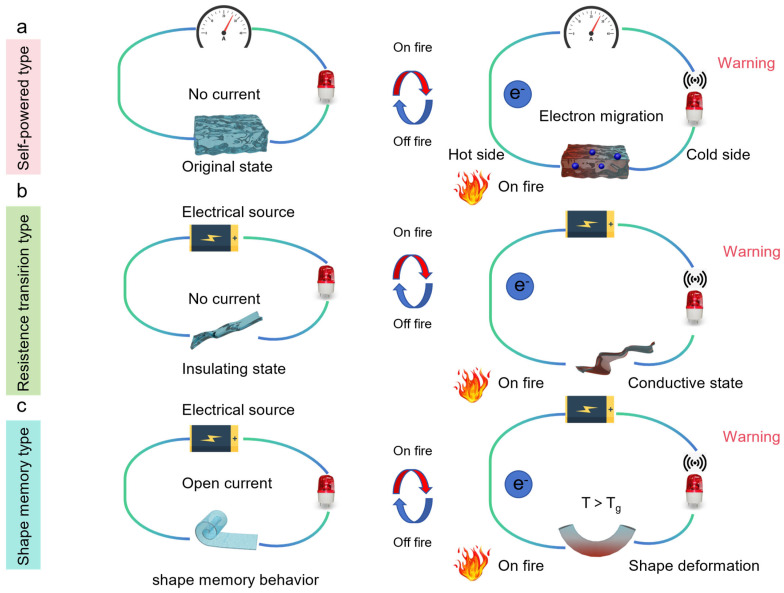
Schematic diagram of fire-warning mechanism. (**a**) Self-powered type, (**b**) resistance transition type, (**c**) shape-memory type.

Fire alarm protection involves deploying sensors and detection devices to swiftly identify early signs of potential fires by responding to factors like smoke, heat, or flames [37,38]. Figure 1 illustrates three main fire alarm system mechanisms: the self-powered type, utilizing nanogenerators and thermoelectric materials; the resistance transition type based on heat induction; and the shape-memory type, relying on shape-memory materials. In the self-powered category, the thermoelectric effect allows graphene-like 2D nanomaterials to convert heat energy into electrical energy when heated. Electrons migrate along temperature gradients to generate electrical signals, resulting in a self-powered fire-warning response. As for the resistance transition type, it primarily comprises a low-voltage electrical source, an alarm lamp, and a fire-warning material, initially in an electrically insulating state. Upon heating, the electrical resistance undergoes a significant change due to polymer carbonization, forming an electrically conductive network. Regarding the shape-change sensor, its mechanism is as follows: the mechanical state of the polymeric materials shifts from a curled to a stretched state upon heating to the T_g_, allowing for the circuit to be connected. Upon subsequent cooling to ambient temperature, it reverts to its pre-programmed temporary shape, thereby disconnecting the circuit to disconnect. Fire-warning protection plays a crucial role in providing early awareness and enhancing overall fire safety by facilitating quick responses to potential fire hazards.

## 3. Graphene-like 2D Nanomaterials-Based Flame Retardant Systems

### 3.1. Graphene and Its Derivatives

Graphene, a two-dimensional carbon sheet with single-atom thickness and a 0.142 nm C–C bond length, possesses exceptional surface area and thermal conductivity [39]. These properties confer advantages in terms of elevated thermal/electrical conductivity, impermeability to gases, and flame resistance. During the combustion of a polymeric matrix, graphene serves as a physical barrier, encapsulating combustible substances and forming a stable barrier layer that reduces the release of combustible gases. Consequently, incorporating graphene at extremely low loading (typically less than 5 wt%) significantly enhances the fire safety of a polymeric matrix, along with providing improvements in thermal, mechanical, and electrical properties [40,41,42].

#### 3.1.1. Utilization of Pristine Graphene

Polymeric materials undergo combustion involving fuel, oxygen, heat, and free radical reactions. Achieving flame retardance in polymeric composites involves inhibiting these components or their circulation [43]. Graphene, known for its stability even in flames, significantly improves thermal stability, mechanical properties, and flame retardance upon incorporation into polymers. The layered structure of graphene encourages the formation of dense char layers during decomposition, which act as physical barriers that delay the release of pyrolysis products and hinder heat transfer. The carbon framework of graphene acts through the “labyrinth effect” creating a tortuous path that enhances the exchange pathway for mass and heat between gas and condensed phases, thereby improving fire safety [40]. Moreover, the substantial specific surface area of graphene enables the efficient adsorption of flammable volatile products, impeding their release during the burning process. Additionally, the excellent compatibility and robust interaction between graphene and polymeric molecules establish a three-dimensional network structure within the polymer matrix. The established network proves advantageous in modifying the viscosity and rheological behavior of the polymer, thereby preventing dripping and impeding the release of organic volatiles during combustion [44,45].

Huang et al. [46] have explored how incorporating 3% graphene sheets into poly(vinyl alcohol) reduced flame hazards. The results showed a substantial 49% reduction in peak heat release rate (PHRR), decreasing from 373 to 190 kW/m², along with a notable increase in time to ignition (TTI) from 18 to 33 s. This phenomenon is ascribed to the formation of a dense and uniform char layer by the condensed-phase flame retardant during the burning process. Additionally, Han et al. [47] have found that graphene’s oxidation degrees and exfoliation levels significantly influence the thermal stability and dynamic viscoelasticity of polymers. Graphene and graphene oxides (GOs) promote carbonization on the polymer surface and fillers, actively contributing to char residue formation. Filling with 5% graphene enhances the fire safety of polymer composites.

#### 3.1.2. Graphene-Based Composites Flame Retardants

Graphene, being a potential novel and environmentally friendly material, holds promise for various engineering applications. Previous studies have shown its superior flame-retardant performance compared to other carbon materials. As is well known, graphene oxide contains abundant reactive oxygen, making it of special significance in engineering. Moreover, creating functional graphene-based composites is easily achievable [48].

Huang et al. [49] have devised a multifunctional additive, Sb-Mo/Br-rGo hybrid, leveraging the mechanical enhancement and thermal barrier effects of GO coupled with the flame retardance of bromine and smoke suppression effect of molybdenum–antimony. Through surface functionalization, the hybrid achieves uniform dispersion in the polymer matrix via easy melt blending. Incorporating 5 wt% Sb-Mo/Br-rGO results in a 31% increase in tensile strength and a 73% boost in the elastic modulus of ABS. In addition to significantly enhancing thermal stability, the addition of 5 wt% Sb-Mo/Br-rGO extends the ignition time by 12 s and markedly reduces peak heat release rate (PHRR) and total smoke production (TSP) by 45% and 54%, respectively.

**Figure 2:**
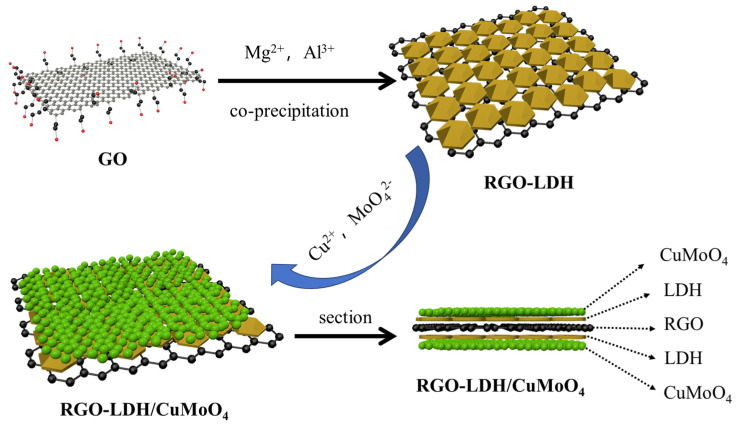
Illustration of the CuMoO_4_ modification of RGO-LDH.

As depicted in Figure 2, Xu et al. [50] have employed the co-precipitation method for the synthesis of a hybrid material that incorporates graphene loaded with magnesium aluminum-layered double hydroxide (RGO-LDH). Subsequently, CuMoO_4_ was applied to the surface, resulting in a modified hybrid denoted as RGO-LDH/CuMoO_4_. The resulting epoxy (EP) composites exhibited enhanced carbon yields, limiting the oxygen index (LOI) and UL-94 vertical combustion grade and thereby enhancing flame retardance.

#### 3.1.3. Molecule-Modified Graphene Composite Flame Retardant

Molecules, such as polymeric and inorganic acid molecules, can be attached to modified graphene sheets to enhance their flame-retardant properties. These modifications involve incorporating additional molecules or chemical groups onto the graphene surface, improving layer dispersion and restraining aggregation, thereby increasing its dispersion and compatibility with polymers. Modified graphene acts as an effective additive in flame retardant formulations, contributing to enhanced mechanical properties, thermal stability, and fire safety in polymer matrices by reducing heat release rates, improving char formation, and inhibiting combustion processes. The specific molecules used for modification can vary and may include compounds designed to enhance flame resistance and other desired properties in the resulting composite materials.

Feng et al. [51] have conducted a study wherein polypropylene was integrated onto graphene oxide sheets (PP-g-GOs) to enhance compatibility in polymer blends containing polyolefins. PP-g-GOs served as versatile fillers, enhancing compatibility, flame retardancy, and thermal strength in PP/PPO blends. The flame-retardant impact of PP-g-GOs coupled with the excellent dispersion of the polymer matrix resulted in a substantial enhancement in flame retardance. Specifically, the addition of 1.5% PP-g-GOs to PP resulted in a reduction in the PHRR value from 1204 in pristine PP to 788 W·g^−1^.

**Figure 3:**
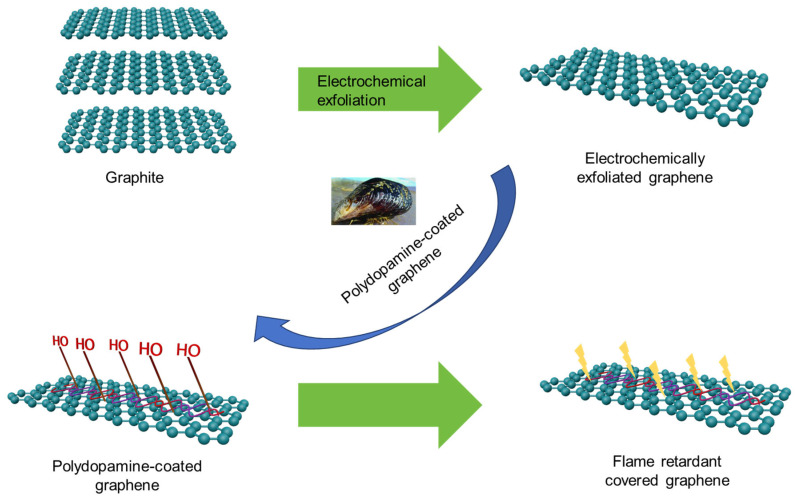
Mussel-inspired functionalization of electrochemically exfoliated graphene.

The fire and smoke suppression potential of graphene in polymer composites is hindered by mass production challenges and inadequate interfacial interactions. Despite electrochemical preparation offering a mass production solution, exfoliated graphene lacks a strong bonding with polar polymer chains. Cai [52] and colleagues have addressed this by successfully using the mussel-inspired functionalization of electrochemically exfoliated graphene and incorporating it into a TPU matrix with polar characteristics (Figure 3). Their results have demonstrated that incorporating 2.0 wt% f-GNS resulted in a notable decrease of approximately 59.4% in the PHRR, 27.1% in THR, 31.9% in the specific extinction area (SEA), and 26.7% in the smoke production rate (SPR) for TPU composites.

### 3.2. MXene and Its Derivatives

In addition to graphene, scholars have extensively researched the modification of polymer flame retardant properties by MXenes. MXenes is a 2D material family discovered in 2011 by Yury’s group [53] and has the universal structure Mn^+1^X_n_T_x_, where M is the transition metal (e.g., Ti, Zr, Cr), X is carbon/nitrogen, T is surface terminations (e.g., O, OH, F, Cl), and n is 1, 2, or 3. With their dense layered structure, exceptional electrical conductivity, varied surface groups, and hydrophilic characteristics, MXenes are progressively being employed in energy storage, sensors, photocatalysis, electromagnetic interference (EMI) shielding, and membrane separation applications [54,55,56]. As anticipated, the great potential of MXenes in improving the fire safety of polymer–matrix composites has expanded their application range in polymers.

#### 3.2.1. Utilization of Pristine MXene

As is well known, the synthesis of 2D layered MXene involves selectively etching the metal layers of MAX phases, with M representing a transition metal. The surfaces of MXene feature functional groups like O, OH, and/or F, providing a hydrophilic surface and excellent solubility in aqueous solutions. Consequently, cationic and water-soluble polymers present viable options as suitable matrices for crafting versatile MXene composites. Wang et al. [57] initially investigated the capability of MXene to enhance the flame retardance of PVA/Ti_3_C_2_T_x_ composite film through straightforward mixing. The results revealed the absence of melting droplets, and the composite film of thin PVA/Ti_3_C_2_T_x_ nearly retained its original shape after combustion. These findings strongly support the enhancing impact of pristine MXene on the thermal stability and anti-dripping performance of polymers.

Incorporating flame-retardant coating layers onto the framework signifies a practical and efficient strategy to improve the fire safety of polyurethane foam (PUF) [58]. In the study by Wang et al. [57], a simple procedure involved immersing PUF in a Ti_3_C_2_T_x_ suspension, followed by drying to produce Ti_3_C_2_T_x_-coated PUF. The well-dispersed Ti_3_C_2_T_x_ nanosheets formed dense flame-retardant coatings on the PUF skeleton, significantly augmenting its anti-dripping performance during the combustion process. However, the coating content of the nanofiller tends to be relatively low, owing to the limited interactions between adjacent layers. Addressing this limitation, Yu’s research [59] has introduced oppositely charged chitosan to create a charge attraction between the MXene and the chitosan layers, thereby enhancing their binding force. The resulting Ti_3_C_2_/chitosan coating acted as an excellent protective barrier, effectively preventing smoke generation and markedly decreasing the fire hazards of PUF.

#### 3.2.2. Utilization of Modified MXene

It is noteworthy that MXene nanosheets exhibit a propensity for aggregation, leading to inadequate dispersion and limited interfacial compatibility with polymers, thereby compromising the flame resistance of MXene within polymer composites. When used in isolation, MXene faces challenges in achieving high efficiency for flame retardation across various materials. However, through functionalization with other elements or molecules, MXene-based nanomaterials present a promising avenue for achieving significantly enhanced performance [60]. Additionally, MXene nanosheets demonstrate the capability to mitigate the formation of smoke and toxic gases, including carbon monoxide, ammonia, and organic volatiles during polymer combustion. Recent advancements have seen the synthesis and application of a range of functionalized MXene flame retardant systems that improve the fire safety of polymer.

**Figure 4:**
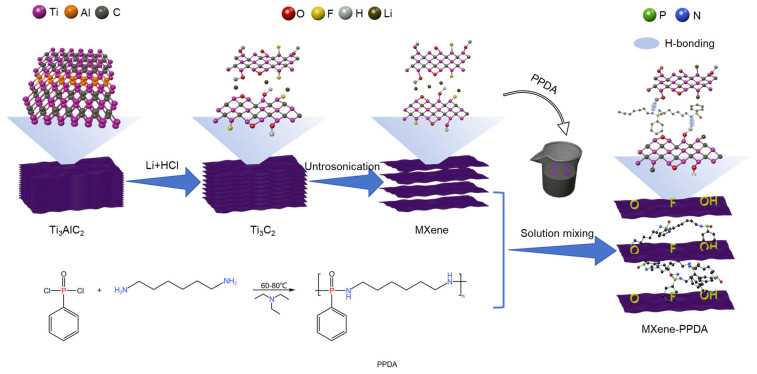
Illustration for the preparation process of MXene-PPDA.

Recently, 2D MXene-based nanomaterial has exhibited significant advantages in enhancing the flame retardance of polymers. Luo et al. [61] conducted a functionalization of MXene with phosphorylated chitosan (PCS) to produce an PCS–MXene hybrid. Subsequently, through solution mixing and hot pressing, the resultant hybrids were incorporated into a TPU matrix. This not only improved the mechanical properties but also significantly enhanced the flame retardance of TPU/PCS–MXene nanocomposites, effectively inhibiting smoke evolution. With the introduction of only 3 wt% PCS–MXene, the composite exhibited a 66.7% reduction in PHRR, a 21.0% decrease in THR, and a 27.7% reduction in total smoke yield in comparison to the original TPU. Xue et al. [62] have illustrated the creation of an innovative MXene-phenyl phosphonic diamino-hexane (MXene-PPDA) nanohybrid by intercalating PPDA into the MXene interlayer (Figure 4). They found that addition of 1.0 wt% MXene–PPDA enabled PLA to attain a UL-94 V-0 rating, with an approximately 22.2% decrease in PHRR, signifying a substantial improvement in flame retardance.

Addressing the significant challenge of developing flame retardant polymer materials with high efficiency and low toxic fume release during combustion, Liu et al. [63] successfully obtained titanium carbide-reduced graphene oxide (Ti_3_C_2_T_x_-rGO) nanohybrids. These nanohybrids were achieved through the hydrogen bonding-induced assembly of Ti_3_C_2_T_x_ and rGO, with applications aimed at enhancing fire safety and the thermal properties of TPU composites. Their findings illustrate the robust adhesion and favorable compatibility of the Ti_3_C_2_T_x_-rGO hybrid with a TPU matrix. With the addition of 2.0 wt% Ti_3_C_2_T_x_-rGO, the TPU nanocomposite exhibited a remarkable 81.2% reduction in the peak SPR and a 54.0% decrease in total smoke release.

#### 3.2.3. Synergism between MXene and Flame Retardants

MXene-based nanomaterials have proven effective as flame-retardant additives in diverse polymer–matrix composites, with greater efficiency and cost-effectiveness resulting from simple blending with synergistic compounds. Typically, the collaborative synergy within polymer–matrix composites results in enhanced thermal stability, reduced heat release rates, and improved char formation compared to the effects of a single component. This promising strategy for developing advanced flame-retardant materials with superior performance involves the amalgamation of MXene with intumescent flame retardants (IFRs) [64,65], phosphorus-containing substances [66], charring agents [67], and other inorganic nanofillers [68].

Lu et al. [69] have explored the synergistic effects of MXene and APP on the fire safety of polyvinyl alcohol (PVA) composite aerogels. The findings reveal that MXene sheets efficiently restricted the release of volatiles from the composites, and the resulting TiO_2_ particles further facilitated the cross-linking and charring of ammonium polyphosphate (APP) and PVA, forming high-quality char barriers. Consequently, the incorporation of just 2.0 wt% MXene significantly enhanced the flame retardancy of PVA/APP composite aerogels.

Biomass materials are frequently employed as char-forming agents in polymer materials designed for fire safety [70]. Lin et al. [71] have developed a ternary structured coating using MXene, phytic acid (PA), and chitosan (CH) to decrease the flammability and smoke release of flexible polyurethane foam (PUF). Leveraging the synergistic charring effects of MXene and biomass materials, the resulting hybrid char layer encapsulated and effectively shielded the unburned PUF during the burning process.

### 3.3. Other Graphene-like 2D Nanomaterials

Graphene-like 2D nanomaterials, including molybdenum disulfide (MoS_2_), boron nitride, and black phosphorene nanosheets, exhibit notable enhancements in flame retardance and reduced smoke toxicity in fire-safe polymer composites [72]. Zhao et al. [73] have achieved the successful growth of magnesium hydroxide (MH) nanoparticles on MoS_2_ nanosheets using a hydrothermal method. Subsequently, the magnesium hydroxy–MoS_2_ hybrid was incorporated into an epoxy resin (EP) matrix. In comparison with the original EP, the resulting EP composite exhibited a reduced rate of thermal decomposition and increased carbon residue. The incorporation of 2 wt% MH-MoS_2_ compounds led to a 27% reduction in peak SPR and a 38% decrease in toxic CO production. Wang et al. [74] have incorporated phthalocyanine zirconium polyphosphazene-functionalized black phosphorene (BP-ZrPZN) as a nano filler in epoxy resin (EP) to create a reinforced system. The results demonstrate a significant enhancement in the smoke-inhibiting capability of black phosphorene with the addition of BP-ZrPZN.

In Dai’s work [75], the authors developed a controllable coating strategy to decorate h-BN with a cyclotriphosphazene-containing boronate polymer (CPBP), forming h-BN-OH@CPBP core–shell nanoplates with varying shell thicknesses. By incorporating h-BN-OH@CPBPs into epoxy resin (EP), they observed that the thermal stability, flame retardancy, and mechanical properties of the resulting nanocomposites depended significantly on the CPBP shell thickness. These nanocomposites achieved a maximum LOI of 28.7%, obtaining a V-0 rating in the vertical burning test at a loading amount of 2.5 wt%. The flexural strengths and modulus were increased by up to 42.12% and 27.85%, respectively, compared to pure EP. Furthermore, the glass transition temperature and dynamic mechanical properties of the nanocomposites were improved.

## 4. Graphene-like 2D Nanomaterials Its Derivatives for Fire-Warning

Given the diverse range of materials available, various types of intelligent fire alarm systems (FASs) have been developed, including those based on graphene oxide [76]. However, the majority of existing fire-warning sensors are designed for indoor applications. Moreover, they often lack reliability and struggle to maintain structural stability in complex conditions. This includes fire alarm systems that utilize smart fire-warning materials based on resistance-type sensing for monitoring sensitive resistance transitions. MXene-based fire-warning sensors outperform traditional fire alarms by offering a swift flame detection response, typically in under 5 s. In comparative fire tests, while standard smoke alarms often take longer than 100 s to respond, MXene-based fire detectors can detect a flame in just 3 s [77,78]. Table 1 provides a comprehensive collection of published fire-warning data pertaining to various material systems. Recent advancements in FASs involve the use of shape-memory polymers for detecting shape transformations, and self-powered FASs that rely on innovations such as nanogenerators and thermoelectric materials.

### 4.1. Self-Powered Fire-Warning Sensors

Self-powered sensor materials operate on the principles of the thermoelectric effect and high electrical conductivity. When subjected to heating, these thermoelectric fire-warning materials can efficiently convert heat energy into electrical energy and provide a self-powered fire-warning response. Pang et al. [86] have devised a self-powered fire alarm system utilizing an innovative multi-layer cylindrical friction electric nanogenerator (MC-TENG). This system harnesses the kinetic energy from moving branches in forests to generate electrical energy for the detection sensor. By incorporating carbon-based micro-supercapacitors alongside MC-TENG, the system proves capable of efficiently, accurately, and reliably reporting fire risks or hazards.

Liu et al. [87] have introduced a self-powered forest fire alarm system (FFAS) integrating thermal and triboelectric effects. This system incorporates a spherical freestanding friction nanogenerator (S-TENG) as the power source, polydopamine-modified graphene oxide (P-GO) as a thermal sensor, and commercial LEDs. FFAS functions as a self-powered early forest fire monitoring and detection system, responding to changes in resistance when exposed to high temperatures or open flames, thereby adjusting the S-TENG output voltage for early warning. Operable without an external power supply, FFAS exhibits a low-temperature response of 160 °C and a rapid response time of 3 s, offering a novel approach for forest fire prevention systems.

### 4.2. Resistance Transition-Type Fire-Warning Sensor 

**Figure 5:**
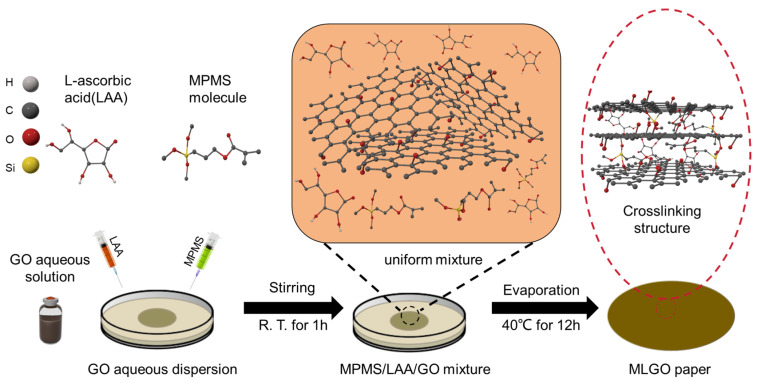
Schematic preparation of MPMS/LAA co-modified GO (MLGO) paper.

Resistance transition-type fire-warning sensors function based on the alteration in electrical resistance when subjected to heat or fire. Typically, the sensor incorporates a material with a distinct transition in resistance at a specific temperature threshold. Zhang et al. [88] have devised and fabricated a paper/coating utilizing environmentally friendly flame-retardant graphene oxide (GO) through a straightforward dual-functionalization approach involving 3-methacryloxypropyltrimethoxysilane and L-ascorbic acid (LAA), as illustrated in Figure 5. This GO network offers an ultra-fast flame detection signal of approximately 1 s. Moreover, it exhibits a low-temperature response speed of up to 120 s, and at a pre-burning temperature of 300 °C, it achieves an exceptionally rapid response time of around 7 s.

In a study conducted by Tang et al. [77], a fire-warning sensor was developed by using MXene decorated with biomimetic polyethylene glycol (PEG) or polyvinyl pyrrolidone (PVP), relying on their hydrogen bonding interactions. When exposed to fire, the resulting MXene paper sensor exhibits a pronounced resistance transition exceeding four orders of magnitude, eliciting a fire-warning response within 2 s. Notably, the sensor demonstrates repeatable fire-warning capability for over 100 cycles while retaining its heightened sensitivity.

### 4.3. Shape-Memory-Type Fire-Warning Sensors

The utilization of shape change as a basis for fire alarms has become a prominent focus in recent years. This concept revolves around the idea that when subjected to elevated temperatures during a fire, shape-memory polymers rapidly transition from a non-permanent shape to a permanent one. This property holds significant promise for diverse applications in the field of fire alarms.

In a study conducted by Wang et al. [89], a straightforward method involving ternary polymerization was introduced to create multifunctional copolyesters derived from common poly(ethylene terephthalate) (PET). This innovative approach incorporates a newly designed third monomer featuring pendant phenylacetylene–phenylimide units. The resulting copolyester exhibits commendable shape-memory and self-healing properties, coupled with exceptional flame retardancy. This research provides a novel perspective for the development of indoor fire alarm systems.

## 5. Concluding Remarks and Future Aspects

The in-depth analysis undertaken in this review has explored the versatile applications of graphene-like 2D nanomaterials. These materials offer the prospect of environmentally friendly flame retardants, contributing to both structural fire protection and fire alarm systems. The urgent need for effective fire protection strategies has been underscored, given the escalating risks posed by polymer materials in various industries. The critical role of flame-retardant materials and advanced fire alarm systems in impeding the rapid propagation of fires and providing early warnings has been emphasized throughout the review. Among these, the utilization of 2D nanomaterials, such as graphene, MXene, molybdenum disulfide and boron nitride has emerged as a promising alternative due to their unique properties, including their high surface area, thermal conductivity, and exceptional flame-retardant performance at low loading contents.

However, a persistent challenge lies in the development of graphene-like 2D nanomaterials with controllable dispersion, heightened flame retardance efficiency, and reduced loading in the polymer matrix. Because of the limited compatibility between graphene-like 2D nanomaterials and the polymeric matrix, these nanosheets have a tendency to restack and aggregate when distributed within a polymer matrix. This tendency can greatly diminish their effectiveness as flame retardants by undermining their physical barrier properties. In this respect, the significance of inorganic-functionalized and molecules-modified graphene-like 2D nanomaterials comes to the forefront. This is due to their fabrication through environmentally benign procedures, which is essential for their widespread application in fire safety polymers. The additional component plays a crucial role in improving the dispersion state of graphene-like 2D nanosheets and strengthening the interface interaction between graphene and the polymer matrix. Simultaneously, both gas-phase and condensed-phase flame retardant mechanisms have been elucidated, emphasizing the importance of disrupting free radical reactions and forming protective char layers. It is important to highlight that additional research is needed to explore the interaction between modifiers and graphene-like 2D nanomaterials, along with a thorough investigation of the precise mechanisms contributing to their good dispersion and synergistic flame-retardant effects.

In the domain of fire-warning mechanisms, the incorporation of 2D nanomaterials, particularly graphene-like nanomaterials, into fire alarm systems has been investigated. These fire alarm systems can be broadly classified into three distinct types: (i) self-powered fire-warning sensors that rely on innovations like nanogenerators and thermoelectric materials; (ii) resistance transition-type fire-warning sensors for monitoring sensitive resistance transitions; (iii) shape-memory-type fire-warning sensors that detecting shape transformations. The exceptional conductivity and large surface area of these materials are identified as key contributors to the improved performance of fire alarm systems, ensuring quicker responses to potential fire incidents. Nevertheless, there is a need for further enhancements in the fire-warning performance and signal transmission of all these fire alarm systems within their potential practical applications due to factors such as high cost, complex processing, uncertain environmental reliability, and poor fire-warning response.

## Figures and Tables

**Table 1:** Comparison of material information and fire alarm response of various material systems.

Composition of Materials	Preparation Method	Fire-Warning Capability	Working Mechanism	Ref.
GO/HCPA (water-soluble multi-amino molecule)	Facile evaporation-induced self-assembly strategy (EISA) method	Ultra-fast fire alarm response time (~0.6 s) and ultra-long alarming period (>600 s)	Resistance transition monitoring	[79]
Silane–GO (graphene oxide) paper	Silane-assisted assembly strategy in water	Flame-detecting response time of ~1.6 s and fire early warning response of ~5 s when attached on a heat resistor	Resistance transition monitoring	[80]
FGO/CNT@PUS (polyurethane sponge)	Layer-by-layer assembly	Coated sponge showed a short alarming time of ~1 s in fire and an early alarming time of ~2 s at 500 °C, along with a super-long alarming period of 2640 s	Resistance transition monitoring	[81]
PVDF-TrFE (poly(vinylidenefluoride-co-trifluoroethylene))/GO/MF (melamine foams)	Layer-by-layer assembly	In high temperature about four seconds to trigger alarm; the nanogenerator can keep the warning working for 22 s after the initial trigger	Thermoelectric response	[82]
ChNCs (chitin nanocrystals)/MXene/ATP (adenosine triphosphate)	Low-temperature evaporation assembly approach	Ultra-fast fire alarm signal of only 0.78 s and an ideal response time of 290 s	Resistance transition monitoring	[83]
PPy-CS (cellulose modified polypyrrole)/MXene	Self-assembly	Triggered a fire alarm in 1.9 s; at second burning, the nanocoating still triggered the fire alarm in 2.3 s	Thermoelectric response	[84]
PMSQ (polymethylsilsesquioxane)/cellulose/MXene	Ice-induced assembly and in situ mineralization	Sensitive fire-warning capability (trigger time was less than 1.8 s)	Resistance transition monitoring	[85]

## Data Availability

Not applicable.

## References

[1] Lian H., Zhang S., Li G., Zhang Y. (2023). Pedestrian Simulation on Evacuation Behavior in Teaching Building of Primary School Emergencies and Optimized Design. Buildings.

[2] Cowled B.D., Bannister-Tyrrell M., Doyle M., Clutterbuck H., Cave J., Hillman A., Plain K., Pfeiffer C., Laurence M., Ward M.P. (2022). The Australian 2019/2020 black summer bushfires: Analysis of the pathology, treatment strategies and decision making about burnt livestock. Front. Vet. Sci..

[3] Yang S., Wang J., Huo S., Wang M., Cheng L. (2015). Synthesis of a phosphorus/nitrogen-containing additive with multifunctional groups and its flame-retardant effect in epoxy resin. Ind. Eng. Chem. Res..

[4] Yang S., Zhang Q., Hu Y. (2016). Synthesis of a novel flame retardant containing phosphorus, nitrogen and boron and its application in flame-retardant epoxy resin. Polym. Degrad. Stab..

[5] Gaan S., Sun G. (2007). Effect of phosphorus and nitrogen on flame retardant cellulose: A study of phosphorus compounds. J. Anal. Appl. Pyrolysis.

[6] Yuan B., Fan A., Yang M., Chen X., Hu Y., Bao C., Jiang S., Niu Y., Zhang Y., He S. (2017). The effects of graphene on the flammability and fire behavior of intumescent flame retardant polypropylene composites at different flame scenarios. Polym. Degrad. Stab..

[7] Velencoso M.M., Battig A., Markwart J.C., Schartel B., Wurm F.R. (2018). Molecular firefighting—How modern phosphorus chemistry can help solve the challenge of flame retardancy. Angew. Chem. Int. Ed..

[8] Song X., Fu T., Pi J., Wang X.L., Song F., Yang Y., Wang R., Deng Z.P., Wang Y.Z. (2023). Bioinspired Machine-Learning-Assisted Early-Fire Perception System Based on VO_2_ Optical Switch. Adv. Funct. Mater..

[9] You C.W., Fu T., Li C.B., Song X., Tang B., Song X., Yang Y., Deng Z.P., Wang Y.Z., Song F. (2022). A Latent-Fire-Detecting Olfactory System Enabled by Ultra-Fast and Sub-ppm Ammonia-Responsive Ti_3_C_2_T_x_ MXene/MoS_2_ Sensors. Adv. Funct. Mater..

[10] Wang X., Kalali E.N., Wan J.-T., Wang D.-Y. (2017). Carbon-family materials for flame retardant polymeric materials. Prog. Polym. Sci..

[11] Lv L.-Y., Cao C.-F., Qu Y.-X., Zhang G.-D., Zhao L., Cao K., Song P., Tang L.-C. (2022). Smart fire-warning materials and sensors: Design principle, performances, and applications. Mater. Sci. Eng. R Rep..

[12] Wu Q., Gong L.-X., Li Y., Cao C.-F., Tang L.-C., Wu L., Zhao L., Zhang G.-D., Li S.-N., Gao J. (2018). Efficient flame detection and early warning sensors on combustible materials using hierarchical graphene oxide/silicone coatings. ACS Nano.

[13] Ma Z., Zhang J., Liu L., Zheng H., Dai J., Tang L.-C., Song P. (2022). A highly fire-retardant rigid polyurethane foam capable of fire-warning. Compos. Commun..

[14] Khan F., Wang S., Ma Z., Ahmed A., Song P., Xu Z., Liu R., Chi H., Gu J., Tang L.C. (2021). A durable, flexible, large-area, flame-retardant, early fire warning sensor with built-in patterned electrodes. Small Methods.

[15] Temane L.T., Orasugh J.T., Ray S.S. (2023). Recent Advances and Outlook in 2D Nanomaterial-Based Flame-Retardant PLA Materials. Materials.

[16] Sharma V., Agarwal S., Mathur A., Singhal S., Wadhwa S. (2023). Advancements in Nanomaterial Based Flame-Retardants for Polymers: A Comprehensive Overview. J. Ind. Eng. Chem..

[17] Ma T., Li L., Pan M., Guo C., Mei C. (2023). Multifunctional MXene-based fire alarm wallpaper with sandwich-like structure for enhanced fire safety and prevention. Chem. Eng. J..

[18] Liang W., Yu B., Wang W., Xiao Y., Yuan Y. (2022). A triazine-based hyperbranched char-forming agent for efficient intumescent flame retardant Poly (lactic acid) composites. Compos. Commun..

[19] Özmen F.K., Üreyen M.E., Koparal A.S. (2020). Cleaner production of flame-retardant-glass reinforced epoxy resin composite for aviation and reducing smoke toxicity. J. Clean. Prod..

[20] Yuan Y., Wang W., Xiao Y., Yuen A.C.Y., Mao L., Pan H., Yu B., Hu Y. (2021). Surface modification of multi-scale cuprous oxide with tunable catalytic activity towards toxic fumes and smoke suppression of rigid polyurethane foam. Appl. Surf. Sci..

[21] Li C., Zhang G., Yuan B. (2023). Exceptional performance of flame-retardant polyurethane foam: The suppression effect on explosion pressure and flame propagation of methane-air premixed gas. Materials.

[22] Wang X., Guo W., Song L., Hu Y. (2019). Intrinsically flame retardant bio-based epoxy thermosets: A review. Compos. Part B Eng..

[23] Zhou X., Qiu S., Mu X., Zhou M., Cai W., Song L., Xing W., Hu Y. (2020). Polyphosphazenes-based flame retardants: A review. Compos. Part B Eng..

[24] Babushok V.I., Deglmann P., Krämer R., Linteris G.T. (2017). Influence of antimony-halogen additives on flame propagation. Combust. Sci. Technol..

[25] Xu W., Wang G., Xu J., Liu Y., Chen R., Yan H. (2019). Modification of diatomite with melamine coated zeolitic imidazolate framework-8 as an effective flame retardant to enhance flame retardancy and smoke suppression of rigid polyurethane foam. J. Hazard. Mater..

[26] Qiu S., Ma C., Wang X., Zhou X., Feng X., Yuen R.K., Hu Y. (2018). Melamine-containing polyphosphazene wrapped ammonium polyphosphate: A novel multifunctional organic-inorganic hybrid flame retardant. J. Hazard. Mater..

[27] Yuan Y., Yang H., Yu B., Shi Y., Wang W., Song L., Hu Y., Zhang Y. (2016). Phosphorus and nitrogen-containing polyols: Synergistic effect on the thermal property and flame retardancy of rigid polyurethane foam composites. Ind. Eng. Chem. Res..

[28] Liu Y., He J., Yang R. (2017). The synthesis of melamine-based polyether polyol and its effects on the flame retardancy and physical–mechanical property of rigid polyurethane foam. J. Mater. Sci..

[29] Shi X., Peng X., Zhu J., Lin G., Kuang T. (2018). Synthesis of DOPO-HQ-functionalized graphene oxide as a novel and efficient flame retardant and its application on polylactic acid: Thermal property, flame retardancy, and mechanical performance. J. Colloid Interface Sci..

[30] Nine M.J., Tran D.N., Tung T.T., Kabiri S., Losic D. (2017). Graphene-borate as an efficient fire retardant for cellulosic materials with multiple and synergetic modes of action. ACS Appl. Mater. Interfaces.

[31] Yuan Y., Shi Y., Yu B., Zhan J., Zhang Y., Song L., Ma C., Hu Y. (2020). Facile synthesis of aluminum branched oligo (phenylphosphonate) submicro-particles with enhanced flame retardance and smoke toxicity suppression for epoxy resin composites. J. Hazard. Mater..

[32] Cai W., Wang B.-B., Wang X., Zhu Y.-L., Li Z.-X., Xu Z.-M., Song L., Hu W.-Z., Hu Y. (2021). Recent progress in two-dimensional nanomaterials following graphene for improving fire safety of polymer (nano) composites. Chin. J. Polym. Sci..

[33] Hajibeygi M., Shabanian M., Omidi-Ghallemohamadi M. (2017). Development of new acid-imide modified Mg-Al/LDH reinforced semi-crystalline poly (amide-imide) containing naphthalene ring; study on thermal stability and optical properties. Appl. Clay Sci..

[34] Yuan Y., Wang W., Shi Y., Song L., Ma C., Hu Y. (2020). The influence of highly dispersed Cu_2_O-anchored MoS_2_ hybrids on reducing smoke toxicity and fire hazards for rigid polyurethane foam. J. Hazard. Mater..

[35] Yuan B., Sun Y., Chen X., Shi Y., Dai H., He S. (2018). Poorly-/well-dispersed graphene: Abnormal influence on flammability and fire behavior of intumescent flame retardant. Compos. Part A Appl. Sci. Manuf..

[36] Yuan Y., Yu B., Wang W. (2022). The influence of poorly-/well-dispersed organo-montmorillonite on interfacial compatibility, fire retardancy and smoke suppression of polypropylene/intumescent flame retardant composite system. J. Colloid Interface Sci..

[37] Carta F., Zidda C., Putzu M., Loru D., Anedda M., Giusto D. (2023). Advancements in forest fire prevention: A comprehensive survey. Sensors.

[38] Zhang G., Liu C., Yang L., Kong Y., Fan X., Zhang J., Liu X., Yuan B. (2024). A flame-retardant and conductive fabric-based triboelectric nanogenerator: Application in fire alarm and emergency evacuation. J. Colloid Interface Sci..

[39] Jamsaz A., Goharshadi E.K. (2023). Graphene-based flame-retardant polyurethane: A critical review. Polym. Bull..

[40] Liu S., Yan H., Fang Z., Wang H. (2014). Effect of graphene nanosheets on morphology, thermal stability and flame retardancy of epoxy resin. Compos. Sci. Technol..

[41] Fu X., Yao C., Yang G. (2015). Recent advances in graphene/polyamide 6 composites: A review. RSC Adv..

[42] Jing J., Zhang Y., Fang Z.-P., Wang D.-Y. (2018). Core-shell flame retardant/graphene oxide hybrid: A self-assembly strategy towards reducing fire hazard and improving toughness of polylactic acid. Compos. Sci. Technol..

[43] Lu S.-Y., Hamerton I. (2002). Recent developments in the chemistry of halogen-free flame retardant polymers. Prog. Polym. Sci..

[44] Chouhan D.K., Rath S.K., Kumar A., Alegaonkar P., Kumar S., Harikrishnan G., Patro T.U. (2015). Structure-reinforcement correlation and chain dynamics in graphene oxide and Laponite-filled epoxy nanocomposites. J. Mater. Sci..

[45] Silva L.C., Silva G.G., Ajayan P.M., Soares B.G. (2015). Long-term behavior of epoxy/graphene-based composites determined by dynamic mechanical analysis. J. Mater. Sci..

[46] Huang G., Gao J., Wang X., Liang H., Ge C. (2012). How can graphene reduce the flammability of polymer nanocomposites?. Mater. Lett..

[47] Han Y., Wu Y., Shen M., Huang X., Zhu J., Zhang X. (2013). Preparation and properties of polystyrene nanocomposites with graphite oxide and graphene as flame retardants. J. Mater. Sci..

[48] Kim F., Luo J., Cruz-Silva R., Cote L.J., Sohn K., Huang J. (2010). Self-propagating domino-like reactions in oxidized graphite. Adv. Funct. Mater..

[49] Huang G., Huo S., Xu X., Chen W., Jin Y., Li R., Song P., Wang H. (2019). Realizing simultaneous improvements in mechanical strength, flame retardancy and smoke suppression of ABS nanocomposites from multifunctional graphene. Compos. Part B Eng..

[50] Xu W., Zhang B., Wang X., Wang G., Ding D. (2018). The flame retardancy and smoke suppression effect of a hybrid containing CuMoO4 modified reduced graphene oxide/layered double hydroxide on epoxy resin. J. Hazard. Mater..

[51] Cao Y., Feng J., Wu P. (2012). Polypropylene-grafted graphene oxide sheets as multifunctional compatibilizers for polyolefin-based polymer blends. J. Mater. Chem..

[52] Cai W., Wang J., Pan Y., Guo W., Mu X., Feng X., Yuan B., Wang X., Hu Y. (2018). Mussel-inspired functionalization of electrochemically exfoliated graphene: Based on self-polymerization of dopamine and its suppression effect on the fire hazards and smoke toxicity of thermoplastic polyurethane. J. Hazard. Mater..

[53] Naguib M., Kurtoglu M., Presser V., Lu J., Niu J., Heon M., Hultman L., Gogotsi Y., Barsoum M.W. (2011). Two-dimensional nanocrystals produced by exfoliation of Ti_3_AlC_2_. Adv. Mater..

[54] Alhabeb M., Maleski K., Anasori B., Lelyukh P., Clark L., Sin S., Gogotsi Y. (2017). Guidelines for synthesis and processing of two-dimensional titanium carbide (Ti_3_C_2_T_x_ MXene). Chem. Mater..

[55] Naguib M., Barsoum M.W., Gogotsi Y. (2021). Ten years of progress in the synthesis and development of MXenes. Adv. Mater..

[56] VahidMohammadi A., Rosen J., Gogotsi Y. (2021). The world of two-dimensional carbides and nitrides (MXenes). Science.

[57] Li L., Liu X., Wang J., Yang Y., Cao Y., Wang W. (2019). New application of MXene in polymer composites toward remarkable anti-dripping performance for flame retardancy. Compos. Part A Appl. Sci. Manuf..

[58] Yang H., Yu B., Song P., Maluk C., Wang H. (2019). Surface-coating engineering for flame retardant flexible polyurethane foams: A critical review. Compos. Part B Eng..

[59] Lin B., Yuen A.C.Y., Li A., Zhang Y., Chen T.B.Y., Yu B., Lee E.W.M., Peng S., Yang W., Lu H.-D. (2020). MXene/chitosan nanocoating for flexible polyurethane foam towards remarkable fire hazards reductions. J. Hazard. Mater..

[60] Li W., Yin Z., Qi L., Yu B., Xing W. (2023). Scalable production of bioinspired MXene/black phosphorene nanocoatings for hydrophobic and fire-safe textiles with tunable electromagnetic interference and exceeding thermal management. Chem. Eng. J..

[61] Luo Y., Xie Y., Geng W., Dai G., Sheng X., Xie D., Wu H., Mei Y. (2022). Fabrication of thermoplastic polyurethane with functionalized MXene towards high mechanical strength, flame-retardant, and smoke suppression properties. J. Colloid Interface Sci..

[62] Xue Y., Feng J., Huo S., Song P., Yu B., Liu L., Wang H. (2020). Polyphosphoramide-intercalated MXene for simultaneously enhancing thermal stability, flame retardancy and mechanical properties of polylactide. Chem. Eng. J..

[63] Liu C., Wu W., Shi Y., Yang F., Liu M., Chen Z., Yu B., Feng Y. (2020). Creating MXene/reduced graphene oxide hybrid towards highly fire safe thermoplastic polyurethane nanocomposites. Compos. Part B Eng..

[64] Huang H., Dong D., Li W., Zhang X., Zhang L., Chen Y., Sheng X., Lu X. (2020). Synergistic effect of MXene on the flame retardancy and thermal degradation of intumescent flame retardant biodegradable poly (lactic acid) composites. Chin. J. Chem. Eng..

[65] Huang S., Wang L., Li Y., Liang C., Zhang J. (2021). Novel Ti_3_C_2_T_x_ MXene/epoxy intumescent fire-retardant coatings for ancient wooden architectures. J. Appl. Polym. Sci..

[66] Liu C., Yang D., Sun M., Deng G., Jing B., Wang K., Shi Y., Fu L., Feng Y., Lv Y. (2022). Phosphorous-Nitrogen flame retardants engineering MXene towards highly fire safe thermoplastic polyurethane. Compos. Commun..

[67] Luo Y., Xie Y., Jiang H., Chen Y., Zhang L., Sheng X., Xie D., Wu H., Mei Y. (2021). Flame-retardant and form-stable phase change composites based on MXene with high thermostability and thermal conductivity for thermal energy storage. Chem. Eng. J..

[68] Zhou B., Li Y., Li Z., Ma J., Zhou K., Liu C., Shen C., Feng Y. (2021). Fire/heat-resistant, anti-corrosion and folding Ti_2_C_3_T_x_ MXene/single-walled carbon nanotube films for extreme-environmental EMI shielding and solar-thermal conversion applications. J. Mater. Chem. C.

[69] Sheng X., Li S., Zhao Y., Zhai D., Zhang L., Lu X. (2019). Synergistic effects of two-dimensional MXene and ammonium polyphosphate on enhancing the fire safety of polyvinyl alcohol composite aerogels. Polymers.

[70] Yuan Y., Lin W., Xiao Y., Yu B., Wang W. (2024). Flame-retardant epoxy thermosets derived from renewable resources: Research development and future perspectives. J. Mater. Sci. Technol..

[71] Lin B., Yuen A.C.Y., Chen T.B.Y., Yu B., Yang W., Zhang J., Yao Y., Wu S., Wang C.H., Yeoh G.H. (2021). Experimental and numerical perspective on the fire performance of MXene/Chitosan/Phytic acid coated flexible polyurethane foam. Sci. Rep..

[72] Yang L., Xu W., Shi X., Wu M., Yan Z., Zheng Q., Feng G., Zhang L., Shao R. (2023). Investigating the thermal conductivity and flame-retardant properties of BN/MoS_2_/PCNF composite film containing low BN and MoS_2_ nanosheets loading. Carbohydr. Polym..

[73] Zhao S., Yin J., Zhou K., Cheng Y., Yu B. (2019). In situ fabrication of molybdenum disulfide based nanohybrids for reducing fire hazards of epoxy. Compos. Part A Appl. Sci. Manuf..

[74] Qiu S., Yang W., Wang X., Hu Y. (2023). Phthalocyanine zirconium diazo passivation of black phosphorus for efficient smoke suppression, flame retardant and mechanical enhancement. Chem. Eng. J..

[75] Zhang H., Mao J., Li M., Cai Q., Li W., Huang C., Yuan C., Xu Y., Zeng B., Dai L. (2020). Design of h-BN@ boronate polymer core-shell nanoplates to simultaneously enhance the flame retardancy and mechanical properties of epoxy resin through the interficial regulation. Compos. Part A Appl. Sci. Manuf..

[76] Cao C.-F., Yu B., Huang J., Feng X.-L., Lv L.-Y., Sun F.-N., Tang L.-C., Feng J., Song P., Wang H. (2022). Biomimetic, mechanically strong supramolecular nanosystem enabling solvent resistance, reliable fire protection and ultralong fire warning. ACS Nano.

[77] Mao M., Yu K.-X., Cao C.-F., Gong L.-X., Zhang G.-D., Zhao L., Song P., Gao J.-F., Tang L.-C. (2022). Facile and green fabrication of flame-retardant Ti_3_C_2_T_x_ MXene networks for ultrafast, reusable and weather-resistant fire warning. Chem. Eng. J..

[78] Qualey III J.R. (2000). Fire test comparisons of smoke detector response times. Fire Technol..

[79] Cao C.-F., Yu B., Chen Z.-Y., Qu Y.-X., Li Y.-T., Shi Y.-Q., Ma Z.-W., Sun F.-N., Pan Q.-H., Tang L.-C. (2022). Fire intumescent, high-temperature resistant, mechanically flexible graphene oxide network for exceptional fire shielding and ultra-fast fire warning. Nano-Micro Lett..

[80] Huang N.-J., Cao C.-F., Li Y., Zhao L., Zhang G.-D., Gao J.-F., Guan L.-Z., Jiang J.-X., Tang L.-C. (2019). Silane grafted graphene oxide papers for improved flame resistance and fast fire alarm response. Compos. Part B Eng..

[81] Chen Z., Chen W., Liu P., Liu Y., Liu Z. (2021). A multifunctional polyurethane sponge based on functionalized graphene oxide and carbon nanotubes for highly sensitive and super durable fire alarming. Compos. Part A Appl. Sci. Manuf..

[82] Liu C.H., Chen C.C., Guo Z.W., Fuh Y.K., Li T.T. (2023). Self-Powered Fire Alarm System with Layer-by-layer Graphene Oxide/Chitosan Nanocoating of Flame-Retardant Nanofilms. Adv. Mater. Technol..

[83] Ma T., Zhou Q., Li L., Pan M., Guo C., Mei C. (2023). Nacre-inspired intumescent flame retardant bridging network for intelligent fire warning and prevention. Chem. Eng. J..

[84] Xie H., Li K., Nian J., Zheng J., Lai X., Wu W., Su X., Wu Y., Zhang X. (2023). A flexible thermoelectric nanocoating with layered bridged heterostructure for sensitive thermosensation and high fire safety. Compos. Part A Appl. Sci. Manuf..

[85] Zhao Y., Zeng Q., Lai X., Li H., Zhao Y., Li K., Jiang C., Zeng X. (2023). Multifunctional cellulose-based aerogel for intelligent fire fighting. Carbohydr. Polym..

[86] Pang Y., Chen S., An J., Wang K., Deng Y., Benard A., Lajnef N., Cao C. (2020). Multilayered cylindrical triboelectric nanogenerator to harvest kinetic energy of tree branches for monitoring environment condition and forest fire. Adv. Funct. Mater..

[87] Liu W., Wang X., Song Y., Cao R., Wang L., Yan Z., Shan G. (2020). Self-powered forest fire alarm system based on impedance matching effect between triboelectric nanogenerator and thermosensitive sensor. Nano Energy.

[88] Zhang Z.-H., Zhang J.-W., Cao C.-F., Guo K.-Y., Zhao L., Zhang G.-D., Gao J.-F., Tang L.-C. (2020). Temperature-responsive resistance sensitivity controlled by L-ascorbic acid and silane co-functionalization in flame-retardant GO network for efficient fire early-warning response. Chem. Eng. J..

[89] Chen L., Zhao H.-B., Ni Y.-P., Fu T., Wu W.-S., Wang X.-L., Wang Y.-Z. (2019). 3D printable robust shape memory PET copolyesters with fire safety via π-stacking and synergistic crosslinking. J. Mater. Chem. A.

